# Clinical and Microbiological Characteristics of Visceral Leishmaniasis Outbreak in a Northern Italian Nonendemic Area: A Retrospective Observational Study

**DOI:** 10.1155/2016/6481028

**Published:** 2016-11-23

**Authors:** E. Franceschini, C. Puzzolante, M. Menozzi, L. Rossi, A. Bedini, G. Orlando, W. Gennari, M. Meacci, G. Rugna, E. Carra, M. Codeluppi, C. Mussini

**Affiliations:** ^1^Infectious Disease Unit, Azienda Ospedaliero-Universitaria Policlinico Modena, Modena, Italy; ^2^Microbiology Laboratory, Azienda Ospedaliero-Universitaria Policlinico Modena, Modena, Italy; ^3^Istituto Zooprofilattico Sperimentale della Lombardia e dell'Emilia Romagna, Brescia, Italy; ^4^University of Modena and Reggio Emilia, Modena, Italy

## Abstract

*Background*. Visceral leishmaniasis (VL) caused by* Leishmania infantum* is endemic in the Mediterranean area. In the last decades a northward spread of the parasite has been observed in Italy. This paper describes a VL outbreak in Modena province (Emilia-Romagna, Northern Italy) between 2012 and 2015.* Methods*. Retrospective, observational study to evaluate epidemiological, microbiological characteristics, and clinical management of VL in patients referring to Policlinico Modena Hospital.* Results*. Sixteen cases of VL occurred in the study period. An immunosuppressive condition was present in 81.3%. Clinical presentation included anemia, fever, leukopenia, thrombocytopenia, and hepatosplenomegaly. Serology was positive in 73.3% of cases, peripheral blood PCR in 92.3%, and bone marrow blood PCR in 100%. Culture was positive in 3/6 cases (50%) and all the isolates were identified as* L. infantum* by ITS1/ITS2 sequencing. The median time between symptom onset and diagnosis was 22 days (range 6–131 days). All patients were treated with liposomal amphotericin b. 18.8% had a VL recurrence and were treated with miltefosine. Attributable mortality was 6.3%.* Conclusions*. VL due to* L. infantum* could determine periodical outbreaks, as the one described; thus it is important to include VL in the differential diagnosis of fever of unknown origin, even in low-endemic areas.

## 1. Introduction

Visceral leishmaniasis (VL) is a protozoan disease caused by members of the* Leishmania donovani *complex and transmitted by phlebotomine sand flies. VL primarily affects the host's reticuloendothelial system and, without treatment, it can be a life-threatening disease. Symptoms include pancytopenia, fever, and hepatosplenomegaly.

VL is endemic in southern Europe in the Mediterranean area, where it is caused by* L. infantum* and the transmission is mainly zoonotic [1]. However, new foci sustained by exotic* Leishmania* species were recently reported in Europe, such as* L. donovani* in Cyprus [2]. Furthermore, two cases of* L. major*/*L. infantum* hybrids in HIV-positive injecting drug users were detected in Portugal [3]. Therefore, monitoring of species circulating in a given region, by either zymodeme analysis or DNA-based typing, should be integrated into an effective surveillance system.

In Italy, the Tyrrhenian littoral, the southern peninsular regions, and the islands have been considered classical endemic zones for VL. Since the early 1990s, however, a northward spread of the disease to previously nonendemic Italian regions has been observed both in humans and in dogs [4–6]. In particular, Biglino et al. showed a seroprevalence of 7.4% in asymptomatic healthy adults in Piedmont Region [5], while Varani et al. reported a little outbreak of autochthonous human VL cases in the Emilia-Romagna region [6].

In this paper we describe a VL outbreak, which took place in the Modena province (Emilia-Romagna, Northern Italy) between 2012 and 2015, focusing on its clinical and microbiological characteristics.

## 2. Materials and Methods

### 2.1. Design

We conducted a retrospective, observational study including all patients with a VL diagnosis referring to Policlinico Modena Hospital from January 2012 to December 2015. We excluded patients living outside Modena province. Epidemiological, clinical, and microbiological features of VL cases were evaluated.

### 2.2. Case Definitions

VL case was defined according to World Health Organization (WHO) criteria that include the presence of VL clinical symptoms (mainly prolonged irregular fever, splenomegaly, and weight loss) with serologic and/or parasitological confirmation (direct microscopy demonstration of* Leishmania* amastigotes, positive* Leishmania* spp. polymerase chain reaction (PCR), or culture) [7, 8].

Hemophagocytic syndrome was defined according to literature criteria [9].

VL recurrence was defined as a new onset of VL signs and symptoms associated with positivity of* Leishmania *spp. PCR after a negative sample in the 6-month follow-up period.

### 2.3. Laboratory Diagnostic Methods

Anti-*L. infantum* antibodies were assayed using an indirect immunofluorescence test (Leishmania Spot-IF; bioMerieux, Marcy l'Etoile, France) and titers were considered significant if equal or above 1 : 80.

DNA in peripheral and in bone marrow blood was investigated using a nested-PCR performed as described elsewhere [10]. Both immunofluorescence assay tests and molecular methods were performed in the Microbiology Laboratory of Policlinico Modena Hospital.

Bone marrow aspirates were inoculated into Evans' modified Tobie's medium. Cultures were maintained at 25°C; the supernatant was examined for parasite growth by light microscopy every three days and subcultured once a week for 4 weeks before they were reported as negative. Positive cultures were transferred to RPMI-1640 supplemented with 10% fetal calf serum for mass culturing. To identify* Leishmania* species, the ribosomal internal transcribed spacers ITS1 and ITS2 were amplified and sequenced as previously described [10]. To each isolate, ITS genotype was assigned according to the number of variable repeats of the 12 microsatellite regions in ITS1 (four sites) and in ITS2 (eight sites). Sequences obtained were aligned using the CLUSTAL W application of BioEdit version 7.0.8.0. and compared with the sequences described by Kuhls et al. [11].

For morphologic assessment, bone marrow smears were stained with May-Grünwald-Giemsa and examined under the light microscope at high magnification. The microscopic evaluation was considered positive in presence of* Leishmania* amastigotes, either into macrophage cytoplasms or dispersed.

### 2.4. VL Treatment

All patients included in the study were treated with liposomal amphotericin B (L-AmB), with a total dose of 40 mg/kg for HIV-infected patients and 20 mg/kg for all the others according to international guidelines [7, 12, 13]. In particular, patients with HIV infection received 4 mg/kg once daily on days 1–5, 10, 17, 24, 31, and 38; HIV-negative patients received 3 mg/kg of L-AmB once daily on days 1–5, 14, and 21. Patients with VL recurrence in the follow-up period were treated with miltefosine 50 mg TID for 28 days. No short course treatment was performed.

### 2.5. Data Collection and Statistical Analysis

Epidemiological, clinical, microbiological, and treatment characteristics were collected. All patients included in the study had a six-month follow-up period.

Due to the small number of patients enrolled, we performed only a descriptive statistical analysis expressing categorical and continuous variables as frequency (percentage) and median values (range values).

On the basis of calendar date of diagnosis and annual epidemiological data of Modena population [14], incidence rates of VL were calculated.

All statistical analyses have been performed using STATA 13 for Windows (StataCorp ltd., College Station, TX).

### 2.6. Ethics Statement

A corresponding approval from the Modena ethic committee was obtained. The study was performed in accordance with the ethical standards laid down in the 1964 Declaration of Helsinki and its later amendments. Due to the retrospective nature of this study the informed consent was waived. Clinical samples were collected during routine diagnosis and/or follow-up, including no additional invasive procedures. Patient records were anonymized prior to analysis.

## 3. Results

**Figure 1:**
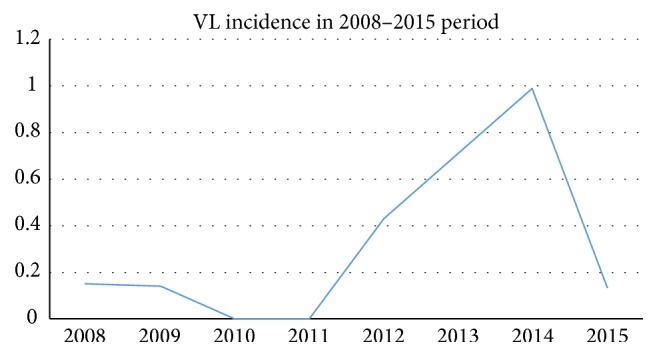
Visceral leishmaniasis incidence per 100,000 inhabitants in 2008–2015 period in the Modena province.

From January 2012 to December 2015, 16 new cases of VL occurred in Modena province (3 patients in 2012, 5 in 2013, 7 in 2014, and 1 patient in 2015). The incidence per 100,000 inhabitants was 0.43 in 2012, 0.71 in 2013, 0.99 in 2014, and 0.14 in 2015.  Figure 1 describes VL incidence in the calendar years from 2008 to 2015 in the Modena province [15].

**Figure 2:**
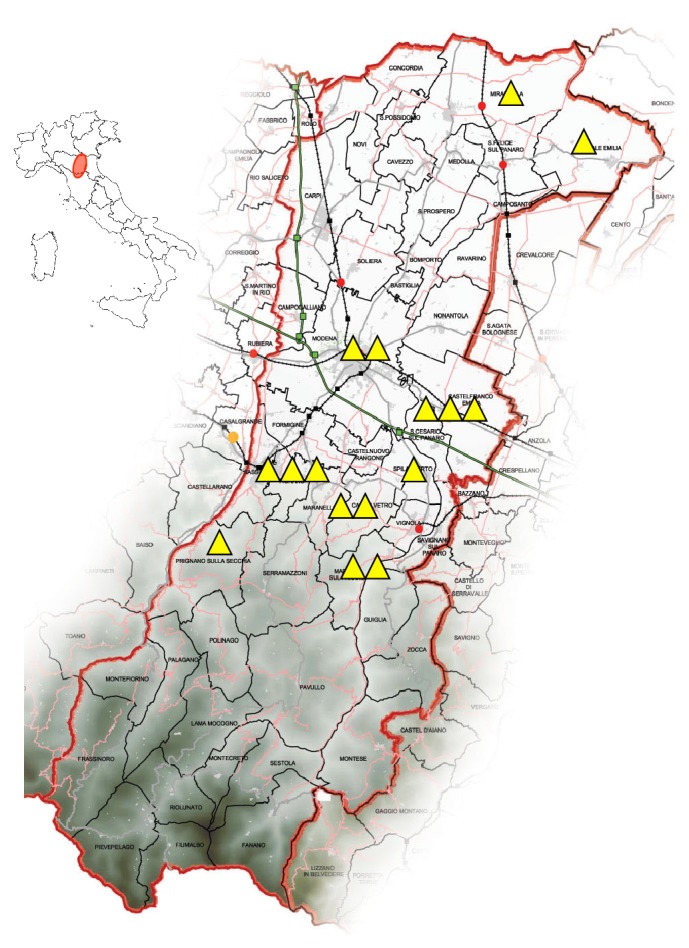
Human visceral leishmaniasis cases distribution map according to residency in Modena province (adapted with permission from www.italomairo.com) from 2012 to 2015. The darkest zones represent the Emilian Apennines.

The majority of patients were resident in rural and hilly areas.  Figure 2 describes VL case distribution. None of the patients have been in endemic areas for human VL in the five years before diagnosis.

81.3% were males (13/16), median age was 63 years (range 33–83 years), and 81.3% had one (9/16) or two (4/16) immunosuppression conditions. In particular, four out of 16 patients (25%) were on chronic steroid therapy, three (18.8%) were HIV-positive, three (18.8%) had a hematologic disease, three (18.8%) had a neoplastic disease, one (6.3%) was an injective drug user, and one (6.3%) was cirrhotic.

Table 1 shows clinical picture at VL diagnosis and microbial tests used for diagnosis of* Leishmania *spp.

The majority of patients (13/16, 81.3%) presented symptoms in autumn/winter. At admission signs and symptoms were fever (15/16, 93.8%), splenomegaly (13/16, 81.3%), asthenia (12/16, 75%), hepatomegaly (11/16, 68.8%), weight loss (6/16, 37.5%), and abdominal lymphadenomegaly (4/16, 25%). Biochemical findings were anemia (16/16, 100%), leukopenia (14/16, 87.5%), thrombocytopenia (13/16, 81.3%), ALT value > 2x upper limit of normal values (7/16, 43.8%), and hemophagocytic syndrome (4/16, 25%). Median baseline creatinine was 1 mg/dL (range 0.8–1.3).

In total, 15 patients performed a serology, 13 patients a peripheral blood PCR, and 12 patients a bone marrow PCR; 6 patients had a culture sample and 11 patients a bone marrow microscopic examination. 11/15 patients (73.33%) had a positive serology and patient 8 had a borderline titer of 1 : 40. Patient 8 was cirrhotic and this could justify the low serologic titer we found. All but one peripheral blood PCR were positive (92.31%), while all the bone marrow PCRs were positive (100%). Three microscopic examination out of eleven (27.27%) demonstrated the presence of amastigotes.

Patient 14 had only a positive serology while no molecular procedures were performed before treatment. The patient was admitted to the hospital with asthenia, anorexia, and hepatosplenomegaly. The biochemical exams showed the presence of pancytopenia and hyperferritinemia. After VL treatment all the signs and symptoms gradually solved as did blood exams. Since the patient had the presence of VL clinical symptoms with serologic confirmation we decided to include her in the analysis (Table 1).

The culture was positive in 3/6 cases. All the isolates were identified as* L. infantum* by the analysis of the 12 microsatellite sites of the ITS1 and ITS2 concatenated sequences [11].

The median time between symptoms onset and hospital admission was 14.5 days (range 4–125 days). The median time between symptoms onset and diagnosis was 22 days (range 6–131 days). The median time between hospital admission and diagnosis was 6 days (range 2–21), with no significant variations during calendar years of the study period.

All patients included in the study were treated with L-AmB as described before. Two patients (12.5%) had a VL recurrence in the follow-up period, both 8 weeks after the first administration of L-AmB. One of the two patients with VL recurrence was on chronic steroid treatment; the other one had a hematological disease. Miltefosine treatment led to clinical and microbiological resolution. One patient died (6.3%) 53 days after symptom presentation, 17 days after diagnosis and starting treatment. He had alcoholic cirrhosis as comorbidity. His death was attributable to VL. L-AmB cure rate was 81.3%.

**Figure 3:**
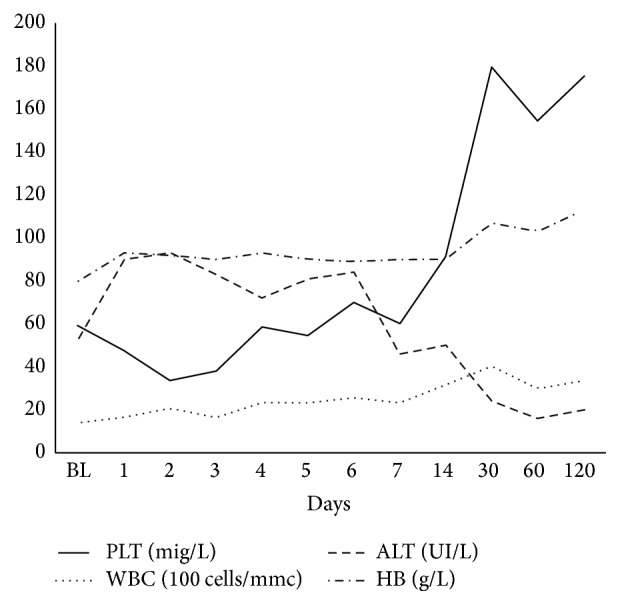
Biochemical trends during visceral leishmaniasis treatment (median values). BL: baseline; PLT: platelets; WBC: white blood cells; ALT: alanine aminotransferase; HB: haemoglobin.

During treatment and follow-up, hematological and biochemical alterations gradually resolved within two weeks as shown in Figure 3. Of note, we did not observe any significant increase of serum creatinine during the observation period despite the use of L-AmB.

## 4. Discussion

Our study describes an outbreak of VL observed in Modena province (Emilia-Romagna region, Northern Italy) from 2012 to 2015. We collected 16 new cases of VL in a confined area with a peak of 0.99 cases per 100,000 inhabitants in 2014, while the average number of cases per year before 2012 was less than 0.2 cases per 100,000 inhabitants [15]. In 2015 the incidence reduces again.

In the early 1970s a severe outbreak of VL was reported in Emilia-Romagna, with a similar distribution of cases, in particular in the foothills of the Apennines zone. However this focus was considered atypical and no further cases were reported in the following 15 years [16].

Although the southern peninsular Italian regions and the islands have been considered classical endemic areas for VL [4], starting from 1990s, also northern continental Italian regions have become focally endemic for* L. infantum* as shown through active surveillance in dogs and phlebotomine sand flies [17]. With regard to human VL, the Italian Ministry of Health reported 230 VL cases among residents in Northern Italy (10.9% of all Italian cases) from 1990 to 2005, mainly from Lombardy (109 cases), Piedmont (42 cases), and Emilia-Romagna (25 cases) [17].

Modena province is a central area of Emilia-Romagna (North-Eastern Italy) (Figure 2), with a mean population of 704,364 inhabitants during the study period [14]. The province covers an area of 2,688 km^2^ and approximately half of it, in its northern part, consists of the Po valley plains, while the remaining part of the province is covered by hills and mountains up to 2,165 m above sea level. Climate is subcontinental, with a mean temperature of 12.4°C and mean annual precipitations of 764 mm [18]. The Po plain, which covers the 48% of the study area, is mainly occupied by agriculture and artificial areas. Hilly and mountainous areas cover the remaining 17% and 35% of the province, respectively, with a prevalence of natural and seminatural areas.

Previous entomological surveys have established the presence of two proven vectors of* L. infantum* in Emilia-Romagna:* Phlebotomus perfiliewi* and* P. perniciosus* [16, 17]. Sand flies are mainly distributed in the hilly areas of the region, including the neighbouring area between Modena and Bologna provinces.* P. perfiliewi* represents the most abundant species [19] and it is considered a less efficient vector of* L. infantum *[17] in comparison to* P. perniciosus*. However, Baldelli et al. [20] reported a canine leishmaniasis focus sustained by* P. perfiliewi* and its role in the VL transmission also cannot be excluded.

Our results show an increase of the median number of VL per year starting from 2012 with a decrease in 2015. Anamnestic information, microbiological data, and the presence of competent vectors, combined with previous sporadic reports of the disease in the Modena province [16], suggest the presence of an autochthonous stable focus of* L. infantum* in the study area, characterized by a recent upsurge. During the same period, Varani et al. reported a VL outbreak in the neighbouring Bologna province, Emilia-Romagna, with 14 cases diagnosed in six months [6].

The reasons of the leishmaniasis reemergence in Emilia-Romagna are not definitely known. With regard to dogs, recognized as primary reservoir hosts of zoonotic VL, any important changes in the canine leishmaniasis epidemiology were observed. Results of a multiyear surveillance program carried out in public kennels of Emilia-Romagna showed an increase in canine leishmaniasis seroprevalence from 2010 (1%) to 2012 (2.4%) [19]. Afterwards a slight decrease was observed [21], with prevalence values lower than those observed in traditionally endemic regions of central and southern Italy [4].

Even though climate change might affect leishmaniasis distribution both directly and indirectly, the association between the two has been only surmised, with lack of definitive evidence [17, 22]. It is demonstrated that from 2000 to 2009 the median annual temperature in Italy increased by 0.8 Celsius degrees compared to the previous decade but no more recent data are available [23].

Further investigations involving multidisciplinary researchers should be carried out in Emilia-Romagna in order to address important epidemiological questions. Studies should be focused on environmental and climate factors influencing the exposure to sand fly bites. Considering that clinical VL cases usually represent the tip of an “infection iceberg,” the prevalence of asymptomatic human carriers and triggers of clinical disease should also be evaluated.

Even though* L. infantum* is the causative agent of VL in Italy, molecular typing with more discriminant tools and characterization of additional isolates could contribute to better understanding the transmission dynamics between humans, vectors, and animal reservoirs as suggested by Chicharro et al. [24].

Due to the increasing incidence of VL in the last years, we expected to find an improvement in our capability to diagnose VL. In the majority of cases the clinical suspicion is the keystone for a rapid diagnosis and, consequently, a rapid treatment and a better prognosis. Actually, we found no difference in the median time between onset of symptoms and diagnosis in the five years considered in our study.

The clinical characteristics at presentation in our cohort did not differ from the literature, anemia, fever, leukopenia, thrombocytopenia, and hepatosplenomegaly being the most frequent symptoms and signs at diagnosis. Thus, being to include VL in the differential diagnosis of fever of unknown origin.

HIV infection is known to be one of the most important risk factors for VL. Nevertheless, only a small percentage of our patients were HIV-positive while we noticed an important presence of other immunosuppression causes (e.g., chronic steroid therapy, hematologic/neoplastic disease, and cirrhosis), recently associated with the onset of VL [25–27]. With the improvement in treatment strategies for hematological and rheumatological diseases (e.g., patients receiving biological agents [28]), we will probably observe a higher number of immunosuppressed patients; thus it is important for clinicians to consider also VL in the differential diagnosis of infections.

In our experience, PCR, both on peripheral and on bone marrow blood, is a sensitive technique for diagnosis of VL with 92.3% and 100% of positive samples, respectively. A positive PCR on peripheral blood in a patient with clinical suspicion of VL was sufficient for VL diagnosis and permitted us to avoid bone marrow aspiration in different cases. On the contrary, a negative PCR on peripheral blood in a patient with high clinical suspicion of VL necessitates further investigations in order to exclude VL diagnosis.

Regarding treatment, we followed WHO recommendations for VL due to* L. infantum, *based on regional differences and expert opinions [7, 12]. We did not explore short course, high dose regimens such as single dose L-AmB. In our study, standard doses of L-AmB showed a good cure rate and profile of tolerability.

Our study has some limitations: first of all the limited number of cases did not allow us to derive definitive conclusions on sensitivity or specificity of the single diagnostic approach; second we had only cases in adult patients; thus our findings could not be taken as evidence in children. Finally, the majority of patients in the study presented an underlying immunosuppressive condition; although no one reported anamnestic VL diagnosis, it was not possible to completely exclude reactivation episodes instead of acute illnesses. A routine serologic test for VL in immunosuppressed patients could be implemented.

## 5. Conclusions

VL due to* L. infantum* could determine periodical outbreaks; thus it is important to include VL in the differential diagnosis of fever of unknown origin, even in low-endemic areas, especially if pancytopenia and/or hepatosplenomegaly are present. Since the reasons of leishmaniosis periodic reemergence in the Emilia-Romagna region are not definitely known, multidisciplinary epidemiological and molecular studies are needed, in order to elucidate gaps in epidemiological aspects.

## Figures and Tables

**Table 1:** Clinical picture and microbial tests used for diagnosis of *Leishmania *spp.

Patient number	Fever	Hepatomegaly	Splenomegaly	Asthenia	Weight loss	Abdominal lymphadenomegaly	Anemia	Leukopenia	Thrombocytopenia	ALT > 2NV	Hemophagocytic syndrome	Serology	Peripheral blood PCR	Bone marrow blood PCR	Culture sample	Bone marrow microscopy
1	•	•	•			•	•	•	•	•	•	−	+	+	N/A	+
2	•	•	•	•			•	•	•		•	−	+	+	N/A	−
3	•			•			•	•	•			1 : 160	+	+	N/A	+
4	•	•	•	•	•		•	•	•	•	•	1 : 640	+	+	N/A	−
5	•	•	•	•			•	•	•			−	+	+	N/A	N/A
6	•	•		•		•	•	•	•			1 : 160	+	+	N/A	+
7	•	•	•	•	•		•	•	•	•	•	1 : 160	+	+	−	−
8	•	•	•				•	•	•	•		1 : 40	+	N/A	−	N/A
9	•	•	•	•	•		•	•	•	•		1 : 320	+	+	+	−
10	•		•	•			•			•		1 : 160	+	+	+	−
11	•		•				•	•		•		1 : 160	N/A	+	+	−
12	•	•	•	•	•		•	•	•			N/A	N/A	+	−	−
13	•		•		•		•	•	•			1 : 320	+	N/A	N/A	N/A
14		•	•	•			•	•	•			1 : 320	N/A	N/A	N/A	N/A
15	•	•	•	•		•	•	•	•			1 : 80	−	+	N/A	−
16	•		•	•	•	•	•					1 : 320	+	N/A	N/A	N/A

PCR: polymerase chain reaction; N/A: not available; ALT: alanine aminotransferase; NV: normal values.
